# Mechanical Behaviour of Orthodontic Auxiliary Photopolymerisable Resins in Simulated Oral Conditions: An In Vitro Study

**DOI:** 10.3390/dj13020067

**Published:** 2025-01-31

**Authors:** Riccardo Favero, Tommaso Zanetti, Vincenzo Tosco, Riccardo Monterubbianesi, Andrea Volpato

**Affiliations:** 1 Unit of Maxillofacial Surgery, Department of Neurosciences, University of Padua, Via Giustiniani 2, 35121 Padua, Italy; riccardo.favero@unipd.it (R.F.); tommaso-zanetti@outlook.it (T.Z.); 2 Department of Clinical Sciences and Stomatology (DISCO), Università Politecnica delle Marche, 60126 Ancona, Italy; v.tosco@univpm.it

**Keywords:** aligners, orthodontics, photopolymerisable resin, restorative materials

## Abstract

Background: The widespread adoption of clear aligners in orthodontic practice has driven the development of biomechanical devices to improve treatment efficiency. The mechanical properties of these materials play a critical role in determining their clinical performance and efficacy. This study investigates the Young’s modulus of Clear-Blokker^®^ (Scheu Dental), a photopolymerisable resin used for the attachment of clear aligner, and evaluates its mechanical behaviour under different curing times (5 s and 10 s) and environmental conditions (dry storage and immersion in artificial saliva at 37 °C). Methods: Forty-eight cylindrical specimens were prepared and subjected to quasistatic compression tests after 14 days. A multi-factorial analysis of variance (ANOVA) at a significance level of 5% was performed to compare the variances. Results: The results showed that samples immersed in artificial saliva had significantly reduced Young’s moduli compared to samples stored in dry conditions (*p* = 0.0213), while no significant difference was observed between curing times. Conclusions: The results suggest that Clear-Blokker^®^ has mechanical properties comparable to those of clear aligner materials, making it suitable as a biomechanical aid for orthodontic treatment. However, further clinical studies are required to confirm its long-term efficacy in the oral environment.

## 1. Introduction

**Figure 1:**
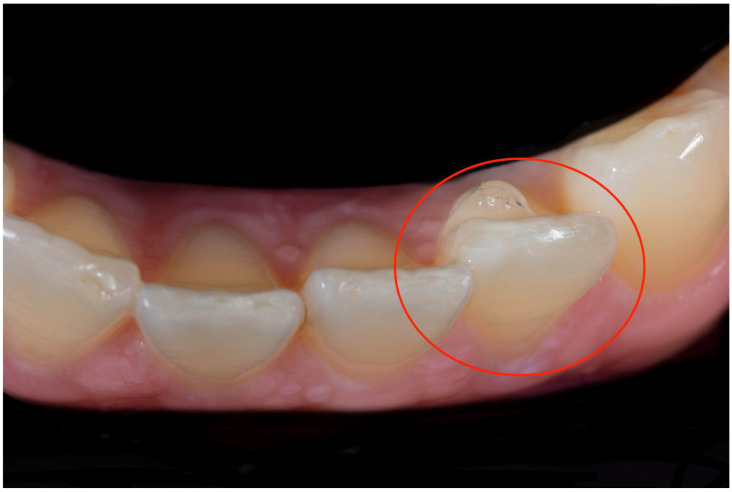
Clinical picture of the “Hill” (red circle).

Orthodontic therapy with clear aligners has expanded rapidly in recent years to meet the growing demand for a comfortable, aesthetic treatment [1,2,3]. Clear aligners generally consist of a series of transparent polymer trays that fit closely over the teeth. These aligners are intended for continuous use by the patient, except during meals and oral hygiene routines, and are typically replaced every one to two weeks to facilitate planned orthodontic tooth movements. With the increased use of the devices, there has been a considerable evolution in the underlying technologies, leading to improved results and expanding the range of application of the treatment [4,5]. Clear aligner therapy does not rely exclusively on the use of aligners: more complex cases can be dealt with using aids such as resin attachments, divots, anchors, precision cuts, bite ramps, and power ridges [6,7]. To remove the potential need for correction where there are discrepancies between the planned and actual tooth movements, or, in any case, to improve the efficiency of the biomechanical system, it has been suggested that a layer of elastic resinous material be applied to the surface of the tooth or teeth to be repositioned (Figure 1). For example, a hill could be placed on the lingual side to generate a buccal movement of the tooth.

The aim of applying this layer is to reactivate the clear aligner and vary the system’s biomechanics. The mechanical characteristics of the material are such that it creates a continuous light force. This biomechanical aid has the name “Hill”. The materials used to manufacture aligners are one of the factors affecting the forces produced during treatment and have been widely studied [8]. Numerous commercial clear aligner systems are available worldwide, with one of their consistent characteristics being the use of clear thermoformed polymer materials during their manufacturing process. While the clinical efficacy of clear aligners may be influenced by a variety of factors, one of the most crucial is the properties of the materials used to manufacture them, as these determine their mechanical and clinical performance. Factors such as water absorption, temperature variations, and cyclic mechanical forces can significantly impact the long-term mechanical properties of aligners. This study evaluates the mechanical properties of Clear-Blokker^®^, particularly its Young’s modulus, under simulated oral conditions. Clear-Blokker^®^ serves as a biomechanical aid that is applied directly to the tooth surface to enhance aligner efficiency by creating an elastic interface. The Young’s modulus, reflecting the material’s rigidity and elasticity, is critical for predicting forces generated during orthodontic treatment. This research investigates variations in Clear-Blokker^®^’s properties, such as polymerisation and degradation, when exposed to factors like artificial saliva and environmental transitions. These analyses aim to provide insights into the material’s performance in clinically relevant conditions. The null hypothesis is that there are no significant differences between the Young’s moduli of samples immersed in artificial saliva and those that were kept dry.

## 2. Materials and Methods

The research project was carried out at the UOC Dental Clinic, University of Padua, Italy, and at the Faculty of Materials Engineering, University of Padua, Italy, in accordance with ISO 604 [9]. In this experimental in vitro study, we examined Clear-Blokker^®^ (manufactured by SCHEU-DENTAL GmbH, Iserlohn, Germany), a light-curing resin (360–420 nm) for dental use with the following chemical composition: 40–70% urethane dimethacrylate (UDMA), 20–50% tricyclodecane dimethanol diacrylate (TCDDMDA), and <10% 1,4-butanediol dimethacrylate (1,4-BDDMA). This material was selected because it is elastic, transparent, mouldable, photopolymerisable, and ergonomic in use. The manufacturer does not provide a complete description of the mechanical characteristics of Clear-Blokker^®^ and there is no published work providing an exploratory evaluation of the mechanical behaviour of the material in an oral environment. This study is the first to evaluate its Young’s modulus. Reusable cylindrical moulds with an internal diameter of 6.3 mm and a height of 6.3 mm were made with a 3D printer. Stoppers were also made of the same material; these were inserted into the base of the moulds to close one end while injecting the resin. Before the casting of each sample, the internal surfaces of the mould and stopper were coated with a uniform layer of Vaseline to prevent the resin from adhering to the mould after polymerisation. Vaseline was used as it does not interfere with the photopolymerisation reaction. The resin was injected into the mould with a syringe fitted with a 0.4 mm diameter cannula, taking care not to let in air bubbles. Excess material was then removed, leaving a flat upper surface. The resin was subsequently polymerised for 5 s or 10 s with a curing light (VALO™ Corded LED curing light; light intensity 1000 mW/cm^2^, wavelength of 385–515 nm). The samples were then extracted from the mould by removing the stopper at the base and gently pushing them out with a plastic cylinder. Any residual material was removed from the mould. The procedure was repeated 48 times by the same operator to obtain 24 samples for the 5 sec light-curing time (5 s) and 24 for the 10 sec light-curing time (10 s). For each time group, 12 samples were sealed in numbered envelopes and kept at room temperature (NB). The other 12 samples were placed in test tubes labelled with identification numbers, then immersed in a water bath (Thermo Scientific™, Waltham, MA, USA, Precision™ GP 02, Dallas, TX, USA) filled with artificial saliva at 37 °C for 14 days to simulate the oral environment (B). The chemical composition of the artificial saliva (pH 6.5) is given in Table 1 [10]. A period of 14 days was considered sufficient for the evaluation, since the absorption of fluids by plastic materials occurs mainly in the first 72–168 h [11,12,13].

**Figure 2:**
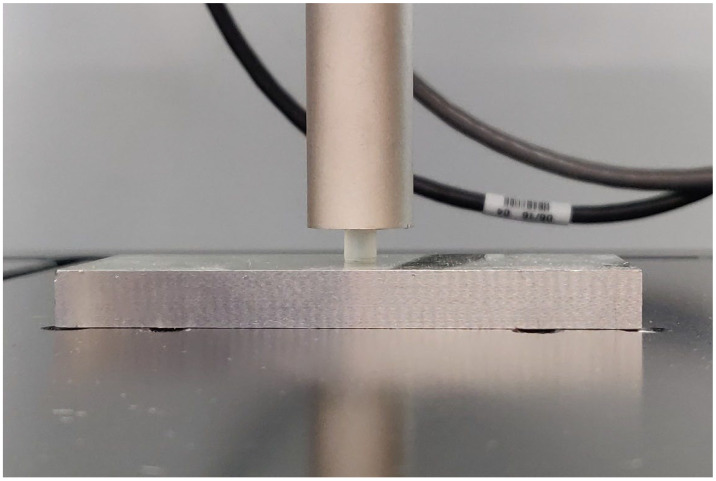
Setup for the compression test on a cylindrical sample of Clear-Blokker^®^.

After 14 days, the diameter and height of the samples were measured with a digital calliper with a precision of ±0.01 mm. Each sample was subjected to a quasistatic compression test at room temperature using an MTS Acumen 3 electrodynamic test system (MTS Systems Corporation, Eden Prairie, MN, USA) with a 3 kN load cell (Figure 2).

A data acquisition frequency of 5 Hz and a deformation rate of 1 mm/min were set for the tests. The yield strength was calculated at 0.2% offset by the straight-line method. The tests were stopped close to the mechanical limit of the machine, which was approximately 2400 N, 83 MPa. Stopping the tests did not affect the calculation of the Young’s modulus and the yield point of the material.

The machine produced data on the force applied (N), the displacement (mm) and the time elapsed (sec) for each sample.

**Figure 3:**
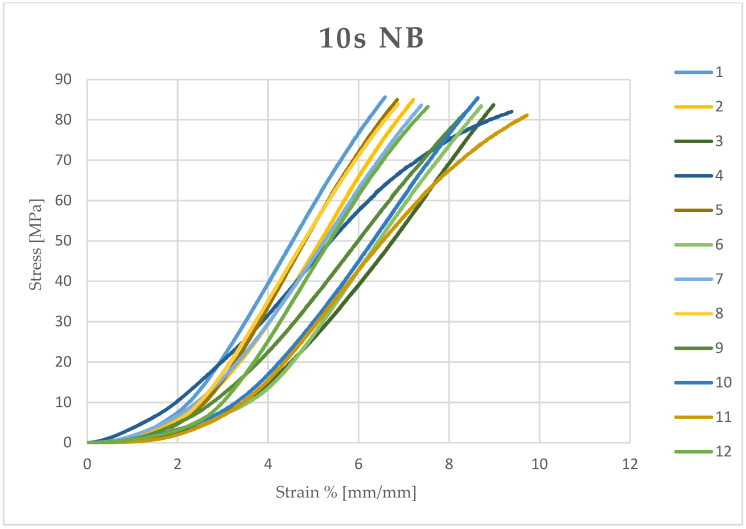
Stress–strain curve of each sample (12) light-cured for 10 seconds (10 s) and kept in a dry environment (NB).

**Figure 4:**
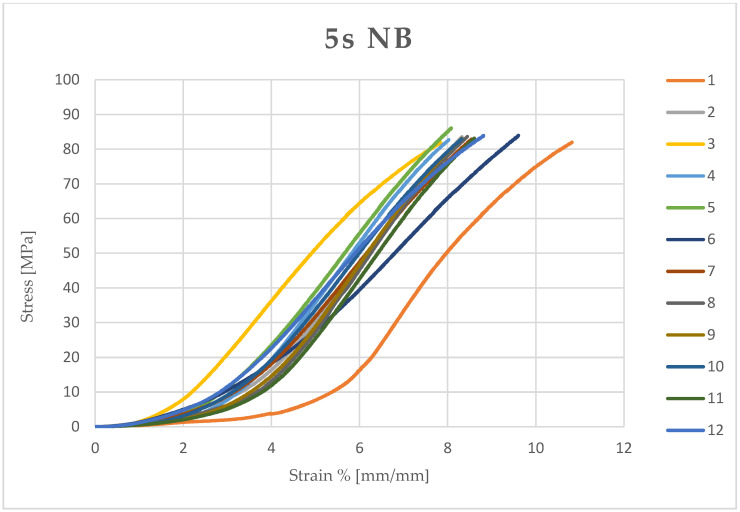
Stress–strain curve of each sample (12) light-cured for 5 seconds (5 s) and kept in a dry environment (NB).

**Figure 5:**
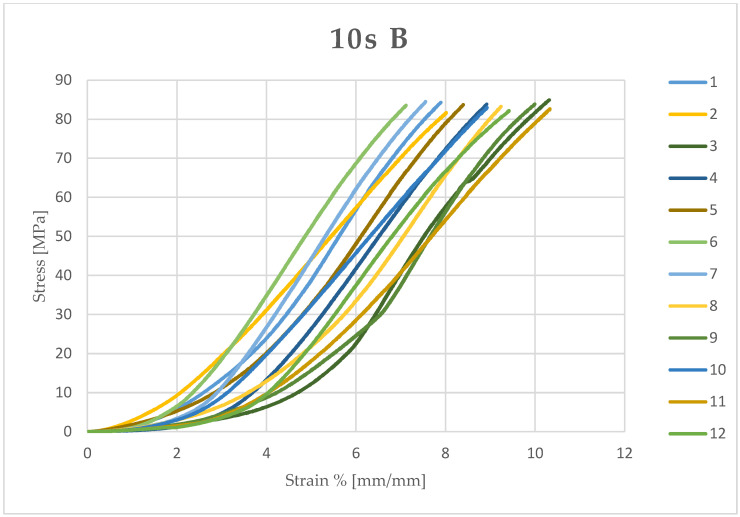
Stress–strain curve of each sample (12) light-cured for 10 seconds (10 s) and kept in an artificial saliva bath (B).

**Figure 6:**
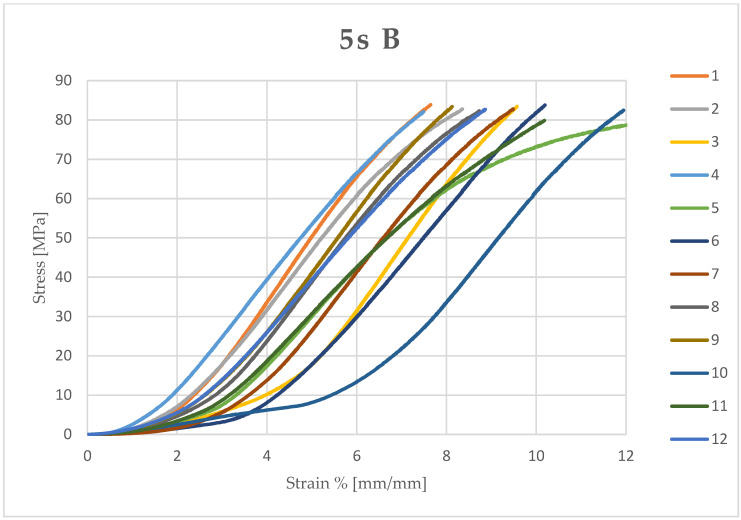
Stress–strain curve of each sample (12) light-cured for 5 seconds (5 s) and kept in an artificial saliva bath (B).

Microsoft Excel (Office 2021) was used to calculate the stress (MPa) and compressive strain (mm/mm) of each sample from the data obtained and the measured diameter and height. The stress and compressive strain were used to generate the stress–strain curve of each test (Figure 3, Figure 4, Figure 5 and Figure 6).

We then proceeded to manually select the data interval corresponding to the elastic region of the curve and calculate the interpolating line to obtain the Young’s modulus (MPa). The yield load (MPa) and yield strength (mm/mm) were also calculated.

The study included 48 cylindrical specimens divided into four experimental groups, each with 12 samples, based on the light-curing time (5 s or 10 s) and storage condition (NB and B): 5 s NB Group, 5 s B Group, 10 s NB Group and 10 s B Group.

### Statistical Analysis

The sample size was determined by referencing prior studies on the mechanical properties of dental materials [14,15], ensuring sufficient statistical power to detect significant differences while minimising variability.

All statistical analyses were performed using Statgraphics Centurion 19 software (v. 19.2.02). The normality of the data was confirmed using the Shapiro–Wilk test, and homogeneity of variances was verified with Levene’s test. A two-way analysis of variance (ANOVA, San Francisco, CA, USA) was conducted to assess the effects of curing time and immersion in artificial saliva on the Young’s modulus, followed by Tukey’s post hoc tests for pairwise comparisons. Statistical significance was set at *p* < 0.05. The results are expressed as the mean ± standard deviation (SD), with 95% confidence intervals provided for each group.

## 3. Results

**Figure 7:**
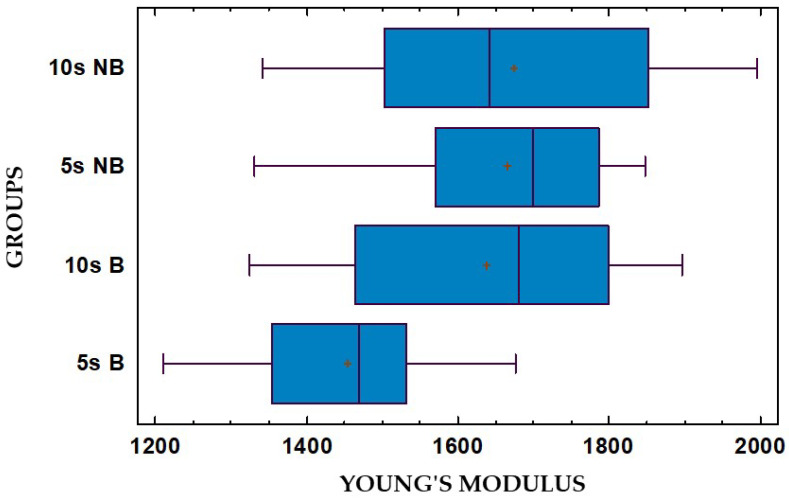
Box plot showing the Young’s moduli (Mpa) of the samples. The samples are subdivided into four groups based on the factors TIME (10 s = 10 sec, 5 s = 5 sec) and BATH (NB = no bath, B = bath). The “+” symbol represents the mean value.

The Young’s modulus values obtained through the compression tests were analysed with statistical analysis software (Statgraphics Centurion 19 v. 19.2.02, ©2023 Statgraphics Technologies Inc., The Plains, VA, USA). We did not consider it necessary to report the statistical analyses for the values of the yield load and the percentage strain at yield, since the magnitude of the forces necessary to reach the yield point of the material is not equal to the forces exerted during orthodontic treatment with clear aligners. The distribution of the measurements was visualised in a box plot diagram (Figure 7).

The means, standard deviations, and ranges of each group were also calculated (Table 2). Note that the 5 s B group has a lower average Young’s modulus than the other three groups. All groups display a certain variability, probably due to the sample production technique (e.g., the uniformity of photopolymerisation, the presence of microscopic bubbles), but they have no outlier points (according to the Dixon test at a significance level of 1%). After checking the normality of the distribution of the dependent variable (Shapiro–Wilk test) and the homogeneity of the variances in the data groups, we compared the variances using a multi-factorial analysis of variance (ANOVA) at a significance level of 5%.

The variables are independent of each other. A multi-factorial ANOVA was performed with the experimentally obtained Young’s moduli acting as the dependent variable. The analysis revealed a statistically significant effect of the factor bath (*p*-value = 0.0213 *), while the effect of the factor time was not significant (*p*-value = 0.0710). It also excluded any significant interaction between the two factors (*p*-value = 0.0960). Table 3 shows the means and confidence intervals of the Young’s moduli of the samples grouped according to the factors examined. As their determination was one of the aims of this study, the values of the averages are particularly relevant.

## 4. Discussion

The present study evaluates the effects of immersion in artificial saliva and light-curing time, and the combination of these two factors on the Young’s modulus of Clear-Blokker^®^. The findings highlight that immersion in artificial saliva significantly reduces the Young’s modulus of Clear-Blokker^®^. This degradation in mechanical properties aligns with the known water absorption and solubility behaviour of dimethacrylate-based resins. Such changes are clinically relevant, as they mimic real-world conditions in which the material interacts with saliva, potentially affecting its long-term performance. The Young’s modulus is a property of a material that expresses the relationship between the axial force applied to it and the resulting deformation. A higher Young’s modulus indicates greater rigidity. The degree of this property is important, as it determines the elasticity of aligners and aids, thereby allowing the forces generated during treatment to be predicted. We consider this a necessary approach to obtain reliable data and to fully understand the behaviour of the material under examination, including following exposure to a simulated oral environment.

The compression tests assess the effects of quasistatic compressive stress on the elastic modulus and mechanical properties of Clear-Blokker^®^. Quasistatic deformation is a process that occurs at a speed that allows the system under examination to adapt to changes and maintain equilibrium throughout the entire test. Ideally, the system passes through an infinite number of equilibrium states between the initial and final states. Since the movements during orthodontic treatment are generally very slow, we consider it appropriate to approximate the Young’s modulus based on a quasistatic model.

Regarding the bath factor, the samples are immersed in artificial saliva (pH = 6.5) at 37 °C, which best reproduces the biochemical conditions in the oral cavity. This allows us to assess any structural modifications determined by the aqueous environment, temperature, and pH. In an aqueous environment, dimethacrylate-based photopolymerisable resins absorb water and release (solubility) the monomers that did not undergo reaction during polymerisation. With glassy polymers, absorption can be described with a dual-modality model: this is based on absorption sites in the polymer matrix that obey Henry’s law on dissolution and absorption sites in the microvoids in the polymer that are regulated by the Langmuir isotherm [16,17]. Polydimethacrylates are cross-linked glassy polymers. The presence of cross-links between the polymer chains generally reduces the permeability of the polymer by the solvent, even though, in exceptional cases, some studies have associated an increase in the concentration of cross-links with greater water absorption [18]. Previous studies have investigated the degree of absorption and water solubility in two of the substances that comprise Clear-Blokker^®^. One reported a water absorption value of 29.46 ± 0.16 (µg/mm^3^) and a solubility of 6.62 ± 0.12 (µg/mm^3^) for UDMA, the main constituent of Clear-Blokker^®^. Another reported the absorption (1.87 ± 0.01%) and solubility (0.22 ± 0.03%) percentages of a resin containing UDMA and TCDDMDA [16], which are both constituents of the material under study. The type of methacrylate inside the resin could also influence the physical and chemical characteristics of the resin. Various studies have found that water absorption has negative effects on resistance to wear, traction and flexion, and on the elastic modulus of the material [19,20,21,22].

A medium with a slightly acidic pH best replicates clinical situations, but the effects on the immersed resin sample take a long time to manifest [23]. However, temperature, instead, statistically significantly increases the water absorption and solubility of dental resins [24].

The results obtained in this study show that the factors considered trigger variations in the mechanical properties. There was a statistically significant difference between samples exposed to the simulated oral environment and those not that were not. The average Young’s modulus of the former was 1546.3 MPa, lower than that of the samples stored dry (1669.7 MPa), and very similar to that of the material most commonly used to produce clear aligners (PET-G, 1870 MPa, stored in an oral-like environment) [25]. The null hypothesis was rejected with a confidence level of 95.0%. (*p*-value = 0.0213 *)

Regarding the time factor, the ANOVA revealed no correlation between the two different light-curing times and the elastic modulus. Varying the light-curing time between 5 s and 10 s had no effect, as there was no statistically significant difference between the Young’s moduli of these sample groups. Since the light-curing time involves varying the amount of light energy transmitted to the resin, it affects the degree of polymerisation obtained and the Knoop microhardness of the samples [26,27]. The Young’s modulus is correlated with Knoop microhardness [28]. The absence of a statistically significant difference may be due to there being too small a difference between the two light-curing times. Depending on the material, the photoinitiator used and the level of irradiation, most of the photopolymerisation reaction takes place in the first 5 s of exposure, and there is only a small degree of polymerisation occurs in the ensuing seconds [29,30,31]. Another possibility is that the results obtained in this study are partly masked by the error accumulated in the experimental phase. Furthermore, the ANOVA did not allow us to exclude the null hypothesis of no significant interaction between the factors under study with a significance level of 5%.

The Young’s modulus data we obtained are as expected. The values most relevant for the clinical application of the material are those related to the samples immersed in a simulated oral environment. The change in the mechanical properties of Clear-Blokker^®^ following immersion in a simulated oral environment is comparable to that reported for materials used for clear aligners [32]. Consequently, Clear-Blokker^®^ is very likely compatible with clinical use in orthodontics. It is our view, therefore, that this transparent material can be use on the surface of teeth that are to undergo orthodontic movement with a transparent aligner. The aligner would thus exert pressure on a surface that is much more elastic (1546.7 MPa) than the surface of the tooth, improving the effectiveness of the orthodontic force.

## 5. Conclusions

The Young’s modulus value of Clear-Blokker^®^, calculated with the factors “light-curing time” and “immersion in artificial saliva”, was slightly lower than the Young’s modulus of PET-G. Immersing Clear-Blokker^®^ samples in a simulated oral environment caused a significant change in their mechanical properties, which was assessed by means of quasistatic compression tests. The average Young’s modulus of the sample stored in artificial saliva was lower than that of the samples stored in dry conditions, very similar to PET-G, and significantly lower than the tooth’s Young’s modulus. This study does not provide sufficient evidence for the effect of curing times to be considered (5 s vs. 10 s) and there was no statistically significant difference between the average Young’s moduli of the sample groups (calculated using quasistatic compression tests). Thanks to its elastic properties, Clear-Blokker^®^ could be a useful biomechanical aid that may improve the effectiveness of the orthodontic force, improving the biomechanical interaction between the aligner and the tooth surface, making it a practical solution to enhance treatment outcomes in complex cases.

## Figures and Tables

**Table 1:** Chemical composition of the artificial saliva used to recreate the biochemical conditions of human saliva [9].

Component	Content (g/L)
NaCl	0.6
KCl	0.72
CaCl_2_·2H_2_O	0.22
KH_2_PO_4_	0.68
Na_2_HPO_4_·12H_2_O	0.856
KSCN	0.06
NaHCO_3_	1.5
C₆H₈O₇	0.03

**Table 2:** Means, standard deviations, minimum values, maximum values, and value ranges of the Young’s moduli (Mpa) of the four groups of samples.

Group	Mean (MPa)	Standard Deviation (MPa)	Minimum (MPa)	Maximum (MPa)	Range (MPa)
10 s NB	1673.6	210.1	1340.6	1996.0	655.4
5 s NB	1665.9	154.6	1331.4	1847.1	515.7
10 s B	1638.1	203.2	1324.3	1897.1	572.8
5 s B	1454.5	137.7	1210.4	1676.7	466.3

**Table 3:** The mean Young’s modulus for each factor and the standard error of each mean, which is a measure of its sampling variability. The two rightmost columns show the 95.0% confidence intervals of each of the means.

Factors	Count	Mean (MPa)	Standard Error (MPa)	Lower Limit (MPa)	Upper Limit (MPa)
Bath					
NO	24	1669.7	36.6	1596.1	1743.4
YES	24	1546.3	36.6	1472.6	1620.0
Time					
5 s	24	1560.2	36.6	1486.5	1633.9
10 s	24	1655.9	36.6	1582.2	1729.5
bath × time					
NO—5 s	12	1665.9	51.7	1561.7	1770.1
NO—10 s	12	1673.6	51.7	1569.4	1777.8
YES—5 s	12	1454.5	51.7	1350.3	1558.7
YES—10 s	12	1638.1	51.7	1533.9	1742.3
OVERALL MEAN	48	1608.0			

## Data Availability

The data presented in this study are available on reasonable request from the first author (R.F.)
